# A Successful Case of Amputation of the Thigh Treated Antiseptically

**Published:** 1877-01-20

**Authors:** Lewis A. Stimson

**Affiliations:** Surgeon to Presbyterian Hospital, New York


### A SUCCESSFUL CASE OF AMPUTATION OF THE THIGH TREATED ANTISEPTICALLY.

By Lewis A. Stimson, M.D., Surgeon to Presbyterian Hospital, New York.

W——, 37 years old, was admitted to the Presbyterian Hospital 13th November, 1876, for a central osteo-sarcoma occupying the lower half of the left femur. Amputation was performed November 21st, as follows, Drs. Van Buren, Detmold, Keyes, and the resident staff of the hospital assisting:

A medium-sized Lister's spray-producer, supplied with a five per cent. solution of carbolic acid, was so placed that its spray would fall directly upon the cut surface. The thigh was washed with a 2½ per cent. solution of carbolic acid, and the instruments placed in a basin supplied with the same. The antero-posterior flap operation through the upper part of the middle third was performed, as Esmarch's bandage having been applied to the leg, and a tourniquet to the femoral artery. The anterior flap, cut rather short on account of the proximity of the tumor, was made from without inwards, the posterior one by transfixion. The artery was found high up in the posterior flap, and a carbolized catgut ligature applied, both ends of which were cut short. All other bleeding points, about fifteen in number, were secured in the same manner.

It had not been thought prudent to carry the elastic bandage over the tumor itself, and the flow of blood from the enlarged distal veins was, therefore, considerable; but the arterial supply was controlled so effectually by Dr. Van Buren, that little or no blood was lost from the upper side. The sciatic nerve was dissected out and cut off high up, so as to save it from becoming imbedded in the cicatrix in case primary union should not be obtained. A carbolized drainage tube, to provide for the ready escape of the first flow of serum, was placed across the bottom of the wound, and the edges of the latter then brought together with silk sutures prepared with carbolized beeswax (about one to five). A strip of "protective" was laid along the wound, the end of the stump covered with a square piece of muslin dipped in the carbolic solution, and eight thicknesses of carbolized guaze, prepared by Caswell & Hazard, but identical with that made in Edinburgh, placed over all and bound down with a roller bandage. A dram of brandy was given hypodermically, and the patient placed in bed and surrounded with hot-water bottles.

The schock of the operation was considerable, and the temperature fell that evening below 97 deg.; but the next day the patient had reacted finely, and since then he has eaten and slept better than for months previously, and has suffered no pain. Before the operation his general condition was bad, his appetite poor, his tongue red and glazed; his temperature rose from 1½ to 2½ degrees every afternoon, and the pain, especially at night, was very severe. The preparatory treatment consisted of careful nourishment and tonic doses of mercury, in accordance with the facts recently established by Dr. Keyes.

During the first twelve days following the operation his temperature never rose above 99¾ deg.; on the thirteenth day it rose to 100¼ deg., returning on the fifteenth to 98½, an excursion due apparently to the appearance of a large carbuncle over the sacrum.

Two weeks after the operation, the entire wound had united by first intention, except at the outer angle, where the drainage tube had been maintained. No bare bone could be felt, and the sinus was not more than 1½ inches deep. All the sutures had been removed, and it is worthy of remark that, although some were left in place fourteen days, there was no suppuration about them. On the tenth day the patient was able to sit up, and on the fifteenth to walk with crutches.

The dressing was renewed the day after the operation and every second day thereafter for ten days, always under the spray, a frequency which was due to my desire to run no risks, and not to the amount of discharge, of which, if we except the first flow of serum, there has been scarcely half an ounce all told.—*N. Y. Med. Rec.*

